# Solid pseudopapillary neoplasm of the pancreas: A case report

**DOI:** 10.1097/MD.0000000000045700

**Published:** 2025-10-31

**Authors:** Marian Bakos, Stefan Durdik, Milan Dubaj, Ivana Stulajterova, Martina Pobeskova, Oleksandr Dobrovanov, Marian Vidiscak

**Affiliations:** a Department of Surgery, Faculty Hospital Nitra, Nitra, Slovakia; b Department of General Surgery, Oncological Institute of St. Elizabeth, Bratislava, Slovakia; c Department of Children and Adolescents, A. Getlik Clinic for Children and Adolescents of Slovak Medical University and University Hospital Bratislava, Bratislava, Slovakia; d Department of General Surgery, 4th Surgical Clinic of the School of Medicine, Comenius University and University Hospital Bratislava, Bratislava, Slovakia.

**Keywords:** case report, neuroendocrine tumor, pancreas, solid pseudopapillary neoplasm, surgical treatment

## Abstract

**Rationale:**

Solid pseudopapillary neoplasm (SPN) of the pancreas, first described in 1959, is a rare low-grade tumor associated with a good prognosis.

**Patient concerns:**

We report the case of a 28-year-old man without significant preexisting disease who underwent abdominal computed tomography for suspected diverticulitis. Subsequently, an incidental finding revealed a pancreas with a homogeneous structure and a hypodense lesion measuring 22 × 19 mm located in the cranial aspect of the pancreatic body, which was visible in the arterial phase.

**Diagnoses, interventions, and outcome:**

It was suspected to be a tumor closely adjacent to a. lienalis, and infiltration of the artery could not be ruled out. Cytological analysis revealed a well-differentiated neuroendocrine tumor. The patient was then admitted to our hospital for surgery. Subsequently, the diagnosis of SPN of the pancreas was confirmed.

**Lessons:**

Differentiating neoplasms from other pancreatic tumors such as neuroendocrine tumors using cytological or biopsy samples can be challenging. Although a laparotomic approach is usually preferred for these tumors, laparoscopic pancreatectomy is associated with reduced intraoperative blood loss, and shorter operative and hospitalization times. Patients with SPNs usually have a good prognosis after surgery, even in cases of local invasion or multiple metastases. More than 95% of patients with solid pancreatic pseudopapillary neoplasms and no infiltration of the surrounding tissues were treated with complete surgical excision.

## 1. Introduction

Solid pseudopapillary neoplasm (SPN) of the pancreas is a rare tumor, accounting for approximately 0.9% to 2.7% of exocrine pancreatic tumors and <10% of cystic pancreatic tumors.^[1]^

The entity was first described in 1959 by pathologist Virginia Kneeland Frantz and therefore it was called as “Frantz tumor” and descriptive terms such as “solid-cystic tumor.”^[2]^ In 1996 it was reclassified by the World Health Organization (WHO).^[1]^ As histopathologic characterization and immunohistochemical and molecular studies evolved, the WHO adopted the term SPN to reflect its characteristic solid and pseudopapillary architecture and to avoid eponymous confusion. The WHO designation also emphasizes the typically low-grade malignant potential and the specific beta-catenin pathway alterations commonly seen in SPN.

No ethnic predilection was observed for these tumors. However, women aged 22 to 28 years are more commonly affected,^[3]^ with a male:female ratio of 1:10. This tumor accounts for approximately 3.9% to 6.6% of cases in men^[4]^ and occurs at an older age, most often between 33 and 38 years. However, it typically exhibits more aggressive behavior and a worse prognosis.^[5]^ Its peak incidence is at approximately 64 years of age.^[6]^ It can also affect children, with an incidence of 6% to 17% of all pancreatic tumors.^[7]^

## 2. Case presentation

This study was reviewed and approved by the ethics committee of Faculty Hospital Nitra (ethical approval number 05/2024), Nitra, Slovakia. The patient provided written informed consent to participate in this study. Written informed consent was obtained from the patient to publish potentially identifiable images or data in this article.

**Figure 1.**
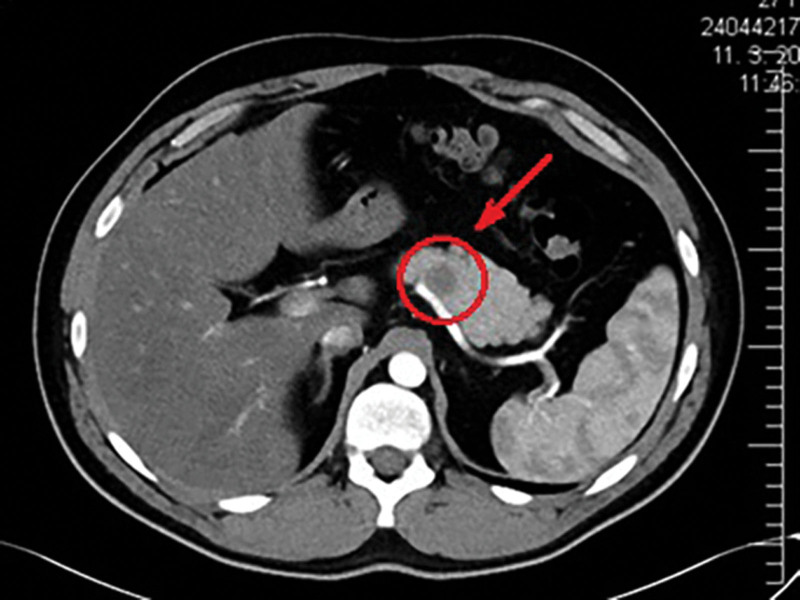
Computed tomography in the axial plane. The red arrow indicates the pancreatic lesion. Source: Authors’ archive, Department of Surgery, Faculty Hospital Nitra, Nitra, Slovakia.

A 28-year-old man with no significant preexisting disease underwent computed tomography (CT) for suspected diverticulitis. Subsequently, an incidental finding revealed a pancreas with a homogeneous structure and a hypodense lesion measuring 22 × 19 mm located in the cranial aspect of the pancreatic body, which was visible in the arterial phase (Fig. 1). It was suspected to be a tumor closely adjacent to a. lienalis, and infiltration of the artery could not be ruled out. The pancreatic duct was not dilated. In the second phase of diagnosis, endoscopic ultrasound-guided fine-needle puncture was performed. The tumor material was applied to 7 slides. Moderate to high cellular smears were identified, consisting of individual and non-cohesive groups of relatively bland, small hyperchromatic cells with oval nuclei and granular chromatin with moderate amounts of eccentric eosinophilic cytoplasm and rosette formation. Cytological results indicated a well-differentiated neuroendocrine tumor (NET).

**Figure 2.**
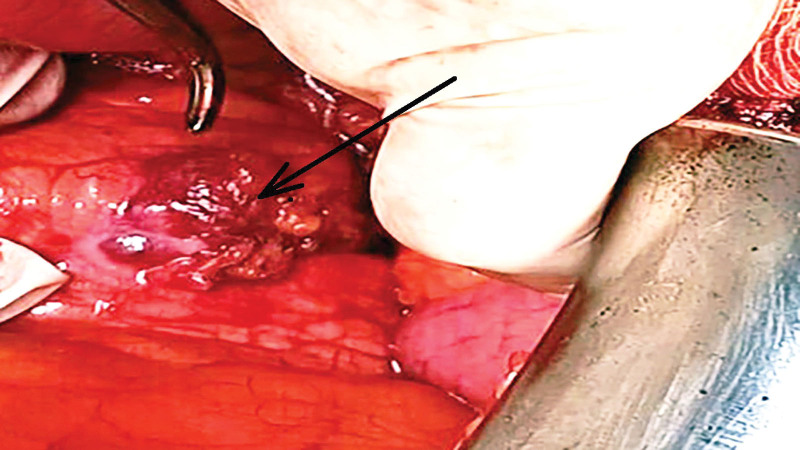
The black arrow points to the pancreatic lesion identified during the surgery. Source: Authors’ archive, Department of Surgery, Faculty Hospital Nitra, Nitra, Slovakia.

**Figure 3.**
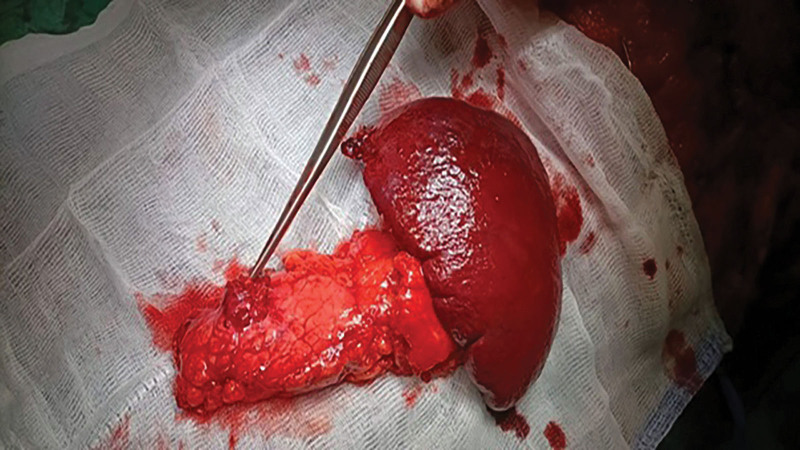
After distal pancreatectomy with splenectomy. Source: Authors’ archive, Department of Surgery, Faculty Hospital Nitra, Nitra, Slovakia.

The patient was then admitted to our hospital for surgery. As part of the preoperative preparation, we administered Clensia and introduced a central venous catheter. The patient subsequently underwent surgery. After preparing the surgical field under general anesthesia, bilateral subcostal incisions were made. The abdominal cavity was surgically opened. While examining the abdominal cavity, we identified a 3 × 2-cm resistance on the anterior wall of the pancreatic body. The resistance infiltrated the omentum from the posterior side; however, the posterior wall of the stomach was not infiltrated. The pancreatic tail and head showed no pathological resistance. a. and v. lienalis were identified. No liver metastasis was observed. The greater curvature of the stomach was mobilized to the diaphragm. We interrupted the vv. gastricae breves using a harmonic scalpel and then ligated it. The spleen was mobilized and the lateral hinge was interrupted using a harmonic scalpel. The upper pole was released from the infiltrated diaphragm, which was then sutured using a Chirlak. We proceeded to an anterior radical antegrade modular pancreatosplenectomy (RAMPS). The peritoneum was opened above the renal fascia at the lower edge of the pancreas. Subsequently, the splenic flexure was released from the lower field of the spleen using a harmonic scalpel. We dissected the superior mesenterica and prepared the neck of the pancreas bluntly. The pancreas was transected using a 3-row linear stapler. We dissected v. lienalis and a. lienalis, which were ligated separately (Figs. 2 and 3). The resistant mass and spleen were sent for perioperative evaluation. The reported result was a NET of the pancreas measuring up to 3 cm with a free resection line. Tissucol glue was then applied to the resected area of the pancreas. A silicone drain (No. 26) was introduced into the left subphrenia, and a similar drain was introduced from the left mesogastrium to the resection line of the pancreas. The abdominal wall was sutured into layers. Total blood loss, even with drapes, was 200 mL.

Postoperatively, we administered a 10% glucose solution with 10 units of insulin. To prevent infection, cefizox and floran were administered. Oral fluid intake, prokinetics, and intermittent parenteral nutrition were administered. We also paid great attention to proper pain management.^[8]^ Once the drainage output was reduced, 1 drain was removed. Subsequently, the gastrointestinal tract was restored. The second drain was shortened and connected to the stoma bag. One complication of the surgery was a fistula that healed with drainage.

## 3. Pathological examination

The definitive histological results showed that the tumor tissue was composed of solid and alveolar structures arranged in relatively uniform medium-sized cells with slightly granular chromatin, indistinct nucleoli, and eosinophilic cytoplasm, with intracytoplasmic hyaline globules in non-accredited special staining (PASD+). A few pseudopapillae with fibrovascular cores and rosette-like structures were also observed. The tumor was interspersed with capillary vascular structures. The stroma was hyalinized, with microdeposits of foamy macrophages and isolated cholesterol crystals with no atypia, mitosis (PHH3), or necrosis. The tumor infiltrated the adjacent pancreatic tissue, and peripheral nerves were partially entrapped in the tumor. Immunohistochemical examination results of the tumor were as follows: beta-catenin+, alpha-1-antitrypsin+, CD10+, synaptophysin focally weak+, INSM1 focally weak +, chromogranin A-, trypsin-, CK7-, CK20-, CEA-, CK19-, CDX2-, S-100-, PAX8-, with a 3% Ki-67 proliferation activity. Within the framework of the differential diagnosis, NET was considered because of the morphological arrangement and cytological characteristics of the tumor cells; however, the extensively examined immunoprofile and the presence of a minor pseudopapillary component in the resected resistant tumor corresponded to SPNs. Seven non-infiltrating lymph nodes were excised. The tumors were pathologically classified as low-grade, pT2, pN0, pMx, LVI0, PN0, or R0, according to the TNM classification. Lipomatosis was also observed in the pancreas and peritumoral hemorrhage and small areas of necrosis were observed in the parenchyma. The spleen was free of tumor changes.

## 4. Discussion

SPN is a rare pancreatic tumor, accounting for about 0.9% to 2.7% of all pancreatic neoplasms. The typical epidemiology is female predominance, often in younger women (2nd–3rd decade), with many cases presenting with nonspecific abdominal pain or mass, sometimes found incidentally.^[1]^

In the retrospective series of 195 SPN cases, mean age was ~31.7 years, with a strong female predominance. A systematic review focusing on male SPN (“Pancreatic SPN in male patients”) collected ~246 male patients. Mean age among males was ~34.3 years (range 4–78). Among male SPN patients, ~35.9% were asymptomatic at diagnosis, despite mean tumor size ≈ 6.3 cm.^[9]^

Presenting symptoms are often vague (abdominal pain/discomfort), or findings are incidental. Rarely, SPNs may present in unusual ways (such as acute pancreatitis) or due to mass effect. For example, the case “A SPN in a man presenting with acute pancreatitis …” describes recurrent pancreatitis episodes with imaging suggestive of pancreatic cancer, only for final histology to reveal SPN.^[10]^ Misdiagnosis is not uncommon. Literature reports SPNs being initially misclassified as pancreatic neuroendocrine tumors (NETs) or other tumor types when cytological, imaging, or immunohistochemical features are ambiguous. For instance, in the “SPN – two unusual cases” paper, 1 case was first diagnosed as a G1 NET, later found to be SPN, with metastases after many years.^[11]^ In addition, small pancreatic neoplasms can be morphologically and cytologically misinterpreted as neuroendocrine neoplasms (NENs) and a biopsy performed at the time of tumor detection cannot always determine whether the lesion represents a NET or an SPN.^[12]^

In our case we identified a well-circumscribed solitary tumor with an average size of 8 to 10 cm and a size range from 0.5 to 35 cm. The tumor was separated from the pancreatic parenchyma using a pseudocapsule. Solid, cystic, hemorrhagic, and necrotic areas were observed within the tumor.

These tumors most commonly occur in the tail and body of the pancreas. In adults, they are more frequently located at the tail, whereas in children, they are often located at the head of the pancreas.^[3]^ Extra-pancreatic localization, such as in the omentum, mesentery, retroperitoneum, ovaries, stomach, or duodenum, rarely occurs.

Distant metastases occur in 7.7% of cases and lymph node metastases occur in 1.6% of cases.^[13]^ Splenic metastases may also occur. Occasionally, these neoplasms can directly infiltrate surrounding structures such as the duodenum, portal vein, or spleen.^[14]^

The etiology of these tumors remains unknown. Possible associations have been described in familial adenomatous polyposis. However, its association with functional endocrine syndromes has not yet been described.^[15]^ The possible association with partial dorsal pancreatic agenesis, a rare congenital anomaly of the pancreas, is currently under investigation.^[16]^

Approximately 15% of patients are asymptomatic and neoplasia is incidentally discovered. Some patients experience palpable resistance or abdominal pain, whereas others present with nonspecific symptoms such as nausea, vomiting, loss of appetite, fever, weight loss, or jaundice. Most symptoms result from the compression of the pancreas by the tumor. Back pain is a less common symptom. Large tumors can present as acute abdomen owing to intratumoral hemorrhage.^[17]^

Ultrasonography, CT, magnetic resonance imaging, and positron emission tomography can be used to detect tumors.

On CT, these tumors appear as well-circumscribed, large, heterogeneous masses with variable ratios of solid-to-cystic components. Solid components are typically located peripherally, whereas cystic components are typically located centrally.^[18]^ On magnetic resonance imaging, this neoplasm appears as a well-defined mass with alternating high and low signal intensities on T1- and T2-weighted images. High T1 signal intensity corresponds to areas of hemorrhagic necrosis, whereas T2 signal intensity varies owing to the presence of hemoglobin degradation products.^[19]^

Ultrasonography-guided fine-needle aspiration, a minimally invasive and well-tolerated procedure, is the diagnostic method of choice. Smears are typically multicellular and form papillary clusters with central fibrovascular nuclei. A selected panel of immunostaining is recommended for a definitive diagnosis to differentiate SPN from NETs (Table 1).^[3]^

**Table 1:** Differential diagnosis of SPN from pancreatic NET (3).

		SPN	NET
Cytology	Cells	Pseudopapillary structures, histiocytes, cholesterol cells, multinucleated giant cells, and macrophages, with positive hyaline globules, large cytoplasm, and vacuoles	Individual cells with round to oval nuclei and granular chromatin
Immunohistochemistry			
Synaptophysin		+	+
Keratin		+	+
CD 56		+	+
Chromogranin		–	+
CD 10		+	–
Vimentin		+	–
β-Catenin		+	–

NET = neuroendocrine tumor, SPN = solid pseudopapillary neoplasm.

Source: Authors’ own elaboration.

If feasible, the treatment of choice is surgery including resection or metastasectomy. However, the tumor is considered unresectable in cases of aortic invasion with > 180° encapsulation of the superior mesenteric artery or vein. Obstruction of the inferior vena cava, unreconstructable portal vein, or occlusion of the superior mesenteric vein also define the lesion as unresectable.

The type of surgery depended on the location and size of the tumor. The common procedures include distal pancreatectomy with splenectomy, distal pancreatectomy with splenic preservation, central pancreatectomy, total pancreatectomy, duodenopancreatectomy, and pylorus-preserving duodenopancreatectomy. The most commonly performed surgery in adolescents is distal pancreatectomy with splenectomy.^[20]^ A Cochrane systematic review compared the effectiveness of the Whipple procedure and pylorus-preserving surgery and did not show any significant differences in the overall morbidity and mortality, except for delayed gastric emptying, which significantly favored the Whipple procedure.

Radiotherapy can be used for unresectable large tumors, as a reduction in tumor size has been reported in some cases. However, adjuvant and neoadjuvant chemotherapy play a role in the treatment. 5-FU and gemcitabine have been used to treat recurrent and metastatic diseases; however, the results are uncertain. Follow-up after complete surgical removal is recommended.^[21]^

The most common complications of surgical treatment include pancreatic fistulae and infections at the surgical site. Other rare complications include bile duct stricture and delayed gastric emptying.^[22]^

## 5. Conclusion

Although SPN is rare and many case reports exist, our case contributes 3 distinctive points.

First, the lesion was initially diagnosed on EUS-FNA cytology as a well-differentiated neuroendocrine tumor (NET), illustrating a recognized diagnostic pitfall when SPNs show focal expression of neuroendocrine markers (e.g., synaptophysin, INSM1) and when material is limited.

Second, the patient is a young man (28 years), whereas SPNs predominantly affect young women; male patients represent a small minority and can present at older ages or with more aggressive features – making. But a review focusing on pancreatic SPN in male patients with 246 male patients found out that mean age among males was 34.3 years, with 26.2% of patients younger than 18 years.^[9]^ Among male SPN patients 35.9% were asymptomatic at diagnosis. A correct preoperative diagnosis (including cytopathology) was provided in 53.6% of patients, with only 40 fine-needle aspiration/biopsies performed. Standard pancreatic resections represented 90.4% of surgical procedures. Although rare, when dealing with a solid-cystic pancreatic mass, even in asymptomatic male patients, a SPN of pancreas should be considered as a possible diagnosis.

Third, we chose the laparotomic approach for our patient for safe and complete removal of the lesion. The pancreatic fistula that resolved with conservative measures and the surgical wound healed completely. Complete surgical removal of the lesion led to disease remission and a good prognosis. The patient is currently regularly followed up by a surgeon and an oncologist.

The operative details and clinical course add practical information for surgeons treating similarly situated small but potentially adherent SPNs.

Patients with SPN usually have a good prognosis after surgery, even with local invasion or multiple metastases. More than 95% of patients with pancreatic SPNs without infiltration of the surrounding tissues are treated with complete surgical excision. A 10-year survival is reported in 96% of the patients. The results of patients with microscopically positive margins and R1 resection were similar to those of patients with extensive resection and negative margins R0. A low Ki-67 index, <5%, indicates slow tumor growth. Several factors predicting patient prognosis and possible tumor recurrence have been investigated, including sex, age, tumor size, margin positivity, presence of distant metastases, perineural invasion, angioinvasion, infiltration of surrounding structures, and Ki-67 proliferative index. Published results are inconsistent, and some are contradictory.^[23,24]^

## Author contributions

**Conceptualization:** Marian Bakos, Oleksandr Dobrovanov, Marian Vidiscak.

**Data curation:** Stefan Durdik, Marian Vidiscak.

**Formal analysis:** Ivana Stulajterova, Martina Pobeskova.

**Funding acquisition:** Marian Bakos.

**Investigation:** Milan Dubaj, Ivana Stulajterova.

**Methodology:** Milan Dubaj, Ivana Stulajterova.

**Project administration:** Stefan Durdik, Marian Vidiscak.

**Resources:** Marian Bakos.

**Supervision:** Stefan Durdik, Marian Vidiscak.

**Validation:** Milan Dubaj, Ivana Stulajterova, Martina Pobeskova.

**Visualization:** Milan Dubaj, Ivana Stulajterova.

**Writing – original draft:** Marian Bakos, Oleksandr Dobrovanov.

**Writing – review & editing:** Marian Bakos, Oleksandr Dobrovanov.
